# Therapeutic Modulation of Cell Morphology and Phenotype of Diseased Human Cells towards a Healthier Cell State Using Lignin

**DOI:** 10.3390/polym15143041

**Published:** 2023-07-14

**Authors:** Mischa Selig, Kathrin Walz, Jasmin C. Lauer, Bernd Rolauffs, Melanie L. Hart

**Affiliations:** 1 G.E.R.N. Center for Tissue Replacement, Regeneration & Neogenesis, Department of Orthopedics and Trauma Surgery, Faculty of Medicine, Albert-Ludwigs-University of Freiburg, Engesserstraße 4, 79108 Freiburg, Germany; mischa.selig@web.de (M.S.); kathrin_walz@yahoo.de (K.W.); jasmin.lauer@uniklinik-freiburg.de (J.C.L.); bernd.rolauffs@uniklinik-freiburg.de (B.R.); 2 Faculty of Biology, University of Freiburg, Schaenzlestrasse 1, 79104 Freiburg, Germany

**Keywords:** lignin, organosolv lignin, cell morphology, cell shape, fibrosis, health, disease, chondrocytes, osteoarthritis, anti-inflammatory

## Abstract

Despite lignin’s global abundance and its use in biomedical studies, our understanding of how lignin regulates disease through modulation of cell morphology and associated phenotype of human cells is unknown. We combined an automated high-throughput image cell segmentation technique for quantitatively measuring a panel of cell shape descriptors, droplet digital Polymerase Chain Reaction for absolute quantification of gene expression and multivariate data analyses to determine whether lignin could therapeutically modulate the cell morphology and phenotype of inflamed, degenerating diseased human cells (osteoarthritic (OA) chondrocytes) towards a healthier cell morphology and phenotype. Lignin dose-dependently modified all aspects of cell morphology and ameliorated the diseased shape of OA chondrocytes by inducing a less fibroblastic healthier cell shape, which correlated with the downregulation of collagen 1A2 (COL1A2, a major fibrosis-inducing gene), upregulation of collagen 2A1 (COL2A1, a healthy extracellular matrix-inducing gene) and downregulation of interleukin-6 (IL-6, a chronic inflammatory cytokine). This is the first study to show that lignin can therapeutically target cell morphology and change a diseased cells’ function towards a healthier cell shape and phenotype. This opens up novel opportunities for exploiting lignin in modulation of disease, tissue degeneration, fibrosis, inflammation and regenerative medical implants for therapeutically targeting cell function and outcome.

## 1. Introduction

Lignin is the second most abundant biopolymer on the planet and is produced in massive amounts as a by-product of the bioethanol, paper and pulp industries. Since less than 2% is used for high value purposes [1,2], lignin’s natural abundance and global availability has spurred new areas of lignin-based research in various biomedical fields over the past decade [3,4]. Yet, how lignin modulates the cell morphology and function of human cells and regulates disease through this modulation has never been assessed.

In nature, lignin is a polyphenolic polymer made up of random linkages that associate with cellulose and hemicellulose [5] and functions as a protective barrier by making biomass resistant to microorganisms and hence, disease. Lignin also greatly enhances the mechanical strength of biomass [6,7]. In human health, lignins have been shown to have diverse therapeutic properties such as antioxidant, anti-microbial and anti-viral effects and for these reasons have been proposed to treat various diseases [3,4,8,9,10]. However, little is known of how lignins can be used to therapeutically target the function of diseased cells towards a healthier phenotype, e.g., through controlling cell morphology [11,12].

Organosolv lignins (OSL) are extracted via the organosolv method, which uses eco-friendly solvents and enzymes and produces a high yield of lignin that is of high quality and purity, making it ideal for use in biomedical and pharmaceutical applications [2]. Fractionation can further decrease lignin’s heterogeneity including the composition of monomers and their linkages as well as functional groups and further standardize its properties [13,14,15,16]. Compared to higher molecular weight (MW) fractions, low MW fractionated lignins are more capable of terminating oxidative chain reactions and oxidative stress due to the increased availability of phenolic hydroxyl groups [14]. Other properties of low MW lignins include anti-inflammatory, anti-elastase [17] and anti-microbial [3] effects. We recently showed that, at a balanced concentration, low MW OSL is non-cytotoxic and biocompatible with major cell types of cartilage (chondrocytes), bone (osteoblasts and bone marrow-derived mesenchymal stromal cells (MSCs), skin (keratinocytes) and oral (periodontal ligament and gingival fibroblasts) tissues [16]. We also showed that the low MW fraction of OSL had increased interfacial adhesion/interactive forces compared to a higher MW fraction of OSL [13,16]. Specifically, the lower MW fraction had more numerous aliphatic hydroxyl functionalities, while having condensed phenolic structures and a less branched conformation as well as an increased hydrogen bonding capacity, which could hypothetically promote intermolecular interactions with cells and thereby modify the phenotype and function of cells towards a healthier phenotype since these characteristics would be expected to increase the availability of the lignin’s functional sites.

Osteoarthritis (OA) is one of the most common degenerative joint diseases and the most frequent cause of physical disability worldwide [18]. To date, no early therapy for this degenerative disease exists and early treatment strategies are needed. In the present study, we used chondrocytes isolated from human diseased OA articular cartilage tissue as a representative diseased cell type to determine if low MW OSL could modulate the cell morphology and phenotype of diseased cells towards a healthier cell state. Chondrocytes are the major resident cell of articular cartilage, the connective tissue that facilitates the movement of joints such as the knee, hip and shoulder and the transmission of mechanical loads applied to those joints during normal everyday activities [19]. Joint trauma (from e.g., sports, accidents or work-related events), abnormal joint mechanics or increased joint load due to increased body weight can damage cartilage and lead to early joint disease characterized by long-term local and circulating low-grade inflammatory and degenerative extracellular matrix (ECM) components produced from the breakdown of cartilage tissue [20]. Importantly, due to cartilage’s avascularity, it has a limited healing and repair capacity. When repair occurs, it generally results in the formation of a fibrocartilage tissue that is biomechanically unstable and frequently undergoes degradation due to the high amounts of collagen type 1 (COL1) produced by chondrocytes that form a weak ECM [21]. This inferior tissue can break down with time, leading to OA [22]. In addition to these phenotypic effects, OA disease development is characterized by major cell morphological changes with chondrocytes acquiring abnormal cell shape characteristics such as a loss of their rounded or spherical morphology in favor of an elongated fibroblast-like cell shape, which correlates with the expression of high levels of unhealthy fibrosis-inducing COL1 and inflammatory-inducing IL-6 and low levels of healthy collagen type 2 (COL2) [23]. Therefore, strategies to modulate the cell morphology and phenotype of diseased chondrocytes towards a healthier cell shape and phenotype would greatly benefit cartilage tissue engineering strategies and also open up many new possibilities for using lignin in an entirely new way.

**Figure 1:**
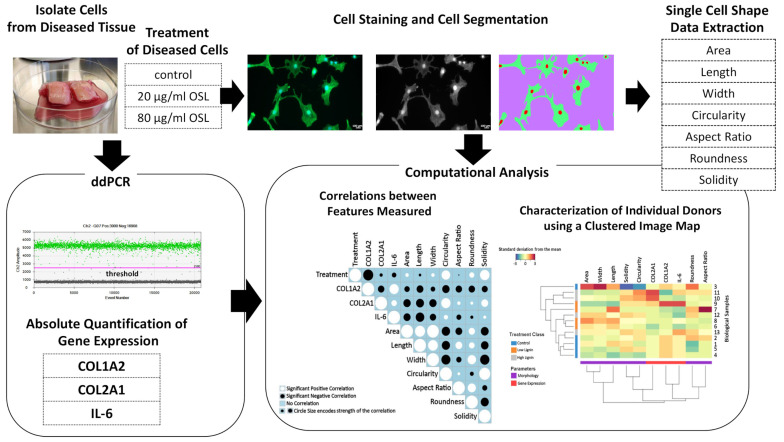
Workflow for determining the effect of lignin on diseased cells. Diseased cells were isolated from human cartilage tissue and treated with lignin. The quantification of gene expression was measured using ddPCR, which allowed absolute quantification of genes in copies/µL by counting the fluorescent positive (green) droplets above the threshold vs. negative (gray) droplets below the threshold. For assessing cell morphology, single cells were detected using image segmentation, and cell shape descriptors were quantified. Both sets of data were assembled for multivariate cell feature analysis. The positive and negative relationship between features under lignin treatment was analyzed using a clustered image map and correlograms.

For the first time, we recently showed that cell morphology could be used as a biological fingerprint for describing healthy, inflamed, and degenerating/diseased chondrocyte phenotype [23]. Since few studies have investigated the uses of OSL for biomedical applications and the effect of lignin on diseased cell morphology, in general, has never been investigated, we combined an automated high-throughput method for quantitatively measuring a panel of cell shape descriptors and absolute quantification of gene expression using droplet digital PCR (ddPCR) to determine whether OSL could modulate the cell morphology and phenotype (COL1, COL2, and IL-6) of inflamed, degenerating human OA diseased cells towards a healthier cell morphology and phenotype. Using an image cell segmentation technique on a large number of diseased chondrocytes, we quantified single cell area, the major (length) and minor (width) axes of the cells, and their aspect ratio, roundness, and the number of cytoplasmic processes depicted as a change in cell circularity and solidity. Combining this data with multivariate data analyses (Figure 1), we investigated the therapeutic potential of lignin in the modulation of cell morphology and phenotype of diseased cells towards a healthier cell state. This is the first study to show that lignin can be used to therapeutically target cell morphology which can change the function of diseased cells towards a healthy cell shape and phenotype. This opens up new possibilities for using lignin in the modulation of disease or tissue degeneration and in tissue engineering strategies.

## 2. Materials and Methods

### 2.1. Preparation of OSL

Organosolv lignin (OSL, Batch No. KO22) derived from beechwood was kindly provided by the Fraunhofer Center for Chemical-Biotechnological Processes (CBP) (Leuna, Germany). The selected method for the fractionating OSL at ambient temperature was based on solvent mixtures of acetic acid and water and fractionated into four different fractions using a sequential precipitation method as previously reported [13,16,24].

The OSL fraction used in this study was previously characterized for structural and physicochemical features and selected based on its high biocompatibility with chondrocytes as well as other cell types commonly used in tissue engineering including human mesenchymal stem cells (MSCs), osteoblasts, periodontal ligament fibroblasts, gingival fibroblasts and keratinocytes [16]. In a step-wise approach, 30% (*w*/*v*) sodium hydroxide (NaOH), which was chosen based on higher cell viability compared to the use of 40% ethanol [16], was added to the low MW OSL fraction to create a stock solution of solubilized OSL. The stock solution was heated to 85 °C while gently stirring and UV sterilized for 30 min.

### 2.2. Isolation of Human OA Chondrocytes from Articular Cartilage and Treatment with OSL

Human OA articular chondrocytes from n = 4–8 different donors were obtained from the medial and lateral femoral condyles of articular cartilage during routine knee replacement surgery with informed patient consent obtained by the Clinic for Department of Orthopedics and Trauma Surgery, University Medical Center Freiburg, Germany, which was conducted according to the guidelines of the Declaration of Helsinki and approved by the Institutional Ethics Committee of the Albert-Ludwigs-University Freiburg (ethics #418/19). These cells were previously characterized [25,26,27]. Under sterile conditions, the cartilage was removed and covered with cartilage explant medium (DMEM low glucose, GlutaMAX supplement, pyruvate, Thermo Fisher Scientific, Schwerte, Germany) containing 10 mM HEPES (Pan Biotech, Aidenbach, Germany), 10% FBS superior, 2% penicillin-streptomycin, 1% amphotericin B, 0.1 mM nonessential amino acids, 0.4 mM L-proline and 0.02 mg/mL L-ascorbic acid phosphate magnesium salt) and incubated for two days at 37 °C and 5% CO_2_. Using 4 mL collagenase XI (1500 U/mL, Sigma Aldrich, St. Louis, MO, USA), 2 mL dispase II (2.4 U/mL, Sigma Aldrich, St. Louis, MO, USA) in 6 mL chondrocyte culture medium, cells were isolated for 6 h at 37 °C and stirred with a sterile magnetic stirring bar at 250 rpm. The digest was filtered through a 100 µm cell strainer (Thermo Fisher Scientific, Schwerte, Germany). The cell pellet was resuspended in media, cultured in a 25 cm^2^ tissue culture flask, and incubated at 37 °C and 5% CO_2_. When the cells were approximately 70% confluent, they were split. After 24 h, passage 1 chondrocytes (9375 cells/cm^2^) were treated with 20 or 80 µg/mL OSL, concentrations previously proven to be non-cytotoxic and biocompatible with chondrocytes [16]. As a control, chondrocytes were cultured in chondrogenic media without OSL. The cells were treated for 6 days with a media change at day 3 with and without OSL. Two identical plates were prepared, one for gene expression analysis and one for high-throughput quantitative single cell morphological analysis.

### 2.3. Droplet Digital PCR for Absolute Quantification of Gene Expression

Ribonucleic acid (RNA) isolation and ddPCR for absolute quantification experiments were performed as previously described in [28]. RNA was isolated using the RNeasy Micro Kit (Qiagen, Hilden, Germany) according to the manufacturer’s protocol. The RNA concentration was determined by measuring the optical density at 260 nm. Then cDNA was synthesized from total RNA with oligo (dT) and random hexamer primers using the Advantage RT-for-PCR Kit (Clontech, Mountain View, CA, USA) according to the manufacturer’s protocol. PCR duplex reactions are performed in 22 µL sample volumes with 11 µL ddPCR Supermix for Probes (no dUTP, Bio-Rad, Hercules, CA, USA), 1.1 µL of each PrimePCR ddPCR Expression Probe Assay (Bio-Rad) labeled with HEX or FAM, 6.6 µL cDNA with 1.5 ng RNA input and 2.2 µL DNase/RNase-free water. Primers and probes specific for human COL1A2, COL2A1 and IL-6 genes were purchased from BioRad. PCR was performed using the QX100 thermal cycler (Bio-Rad) with the following steps. The polymerase activation at 95 °C for 10 min, followed by 40 cycles of denaturation at 94 °C for 30 s and the annealing at 55 °C for 1 min. After cDNA extension, the polymerase was denatured at 98 °C for 10 min and the PCR products were kept at 4 °C until droplet reading. The fluorescence of the droplets was measured by the QX200 Droplet Reader (Bio-Rad) and analyzed using QuantaSoft Software (Version 1.7.4) (Bio-Rad), which quantifies the number of HEX- and FAM-positive and negative droplets and calculates the target concentration for each HEX- and FAM-labeled target gene in copies/µL. Data normalization was achieved using a standardized amount of RNA for reverse transcription and, therefore, a standardized amount of cDNA in the reaction volume.

### 2.4. Cell Staining and Microscopy

To accurately measure single cell morphology, we first stained the chondrocytes after 6 days of incubation with 1 μM calcein (Thermo Fisher Scientific, Schwerte, Germany) and 1 μg/mL Hoechst (Thermo Fisher Scientific, Schwerte, Germany) to visualize the cell body and nucleus. The cells were incubated in the staining solution for 30 min at 37 °C and 5% CO_2_. Then, fresh chondrocyte culture medium was supplied and microscopical images with a 20× magnification were taken with the Axio Observer Z1 microscope (Zeiss, Oberkochen, Germany) in a tile format to image entire cell culture wells.

### 2.5. High-Throughput Quantitative Cell Morphometric Measurements Using a Panel of Cell Shape Descriptors

The bioimage analysis tool QuPath [29] was used to convert large whole image samples to a .tif file format and downsize the images by a factor of three. The images were split into nine single image tiles, and three representative images used for analysis using an in-house Fiji-based [30] single cell shape analysis algorithm and Trainable WEKA Segmentation plugin [31] for segmentation of cells from the background. The WEKA classifier was trained for pixel classification of three classes: nucleus, cytosol, and background. After successfully segmenting the cells from the image background with the WEKA classifier, neighboring cells were separated with a marker-based watershed algorithm. The resulting single chondrocytes of a large number of cells were detected in the binary image maps and single cells were assessed by calculating the following seven cell shape descriptors similar to our previous studies [32,33,34,35]: area of the single cells (µm^2^), major axis [µm] representing cell length; minor axis [µm] representing cell width; circularity (4 × π(area/perimeter^2^); aspect ratio (ratio of major to minor axis), which is used an indicator of cell elongation and different than cell length; roundness (4 × area/(π × major axis length^2^); and solidity (area/convex area (cell)), which measures the density of a cell with a solidity value of 1 representing a solid cell and a value less than 1 representing a cell with an irregular boundary or a cell containing holes.

### 2.6. Correlation Analysis

Correlations were performed using the “R” [36] packages “Hmisc” [37] and “corrplot” [38]. The Spearman Rank Order correlation method was used if one or more variables were categorical. Pearson product-moment was used when variables were numerical. The classes were coded as 0 (control), 1 (20 µg/mL OSL), and 3 (80 µg/mL OSL treatment).

### 2.7. Clustered Image Map (CIM) Allowing Multi-Level Analyses

The CIM was generated using the “mixOmics” [39,40] package in “R” to determine the standard deviation away from the mean on scaled and centered data. This allowed assessment of the relationship between variables and donor variability across all donors in response to treatment.

### 2.8. Statistical Analysis

The data was analyzed using Microsoft Excel (v. 2013) and SigmaPlot v.14.0 (Systat, Chicago, IL, USA). ANOVA on ranks was performed using Dunn’s method as a post hoc test. Statistical differences were considered significant for *p* < 0.05.

## 3. Results

### 3.1. Lignin-Mediated Modulation of Gene Expression in Human Diseased Chondrocytes

**Figure 2:**
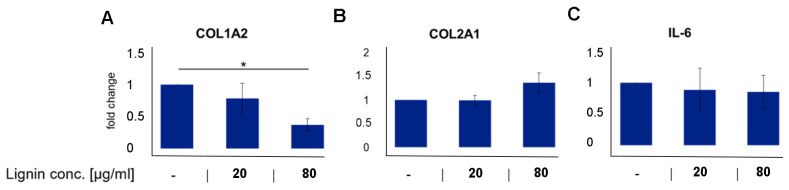
Lignin effects on (**A**) COL1A2 (unhealthy phenotypic marker), (**B**) COL2A1 (healthy phenotypic marker), and (**C**) IL-6 (inflammatory marker) in human diseased cells. OA chondrocytes were treated for 6 days with or without lignin. Data is presented as mean fold change compared to control of n = 4–8 donors per group +/− SEM. * *p* < 0.05.

As a first step, we investigated whether OSL affected the health and inflammatory profile of degenerating OA diseased chondrocytes. Cells were treated with or without OSL for 6 days at biocompatible concentrations with a range of primary human cell types isolated from various tissues [16]. OSL treatment of human OA chondrocytes resulted in a significant dose-dependent decrease in COL1A2 expression, an unhealthy phenotypic marker. It showed a trend in increasing COL2A1, a healthy phenotypic marker of cartilage with 80 μg/mL OSL resulting in a 0.4-fold decrease in the expression of COLA12 and a 0.6-fold increase in the expression of COL2A1 (Figure 2A,B). OSL did not affect the expression of IL-6 (Figure 2C).

### 3.2. Lignin-Mediated Modulation of a Diseased Cell Shape into a Healthier Cell Shape

**Figure 3:**
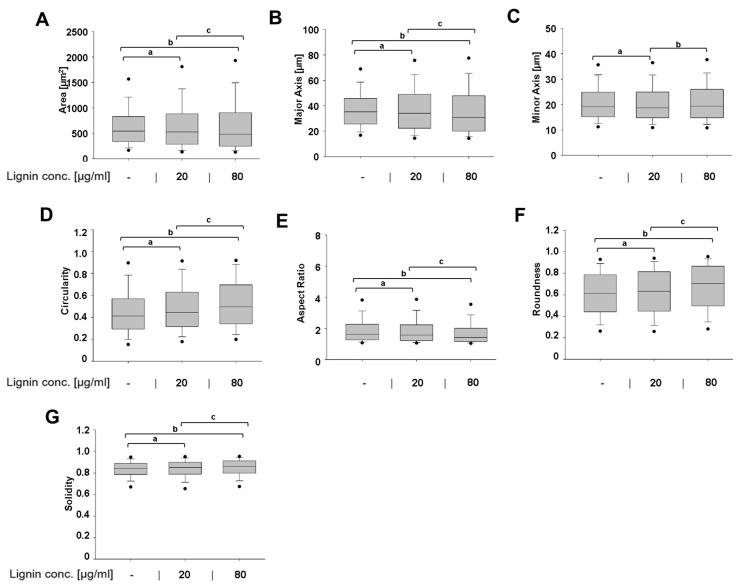
Lignin effects on the cell morphology of human diseased chondrocytes. The following cell morphology descriptors were measured: (**A**) area, (**B**) major axis (length), (**C**) minor axis (width), (**D**) circularity, (**E**) aspect ratio, (**F**) roundness, and (**G**) solidity in OA chondrocytes treated for 6 days with or without lignin. Data is expressed as raw data of n = 4–8 donors per group with 11,726, 6088, and 4495 cells analyzed in control, 20, and 80 µg/mL lignin-treated groups, respectively. The boxplots demonstrate the median (central line) and the data’s 25th and 75th percentile values. The whiskers below and above the box plots represent the 10th and 90th percentile values and the black points illustrate the 5th and 95th percentiles. Significant differences (*p* < 0.05) are indicated as follows: ^a^ between the control vs. 20 µg/mL lignin-treated groups, ^b^ between the control vs. 80 µg/mL lignin-treated groups, and ^c^ between the 20 and 80 µg/mL groups.

Osteoarthritis disease development is characterized by major cell morphological changes. Healthy chondrocytes are typically round or spherical, but as tissue degeneration progresses, chondrocytes acquire an abnormal cell shape with the cells becoming less round/less spherical and more elongated with cells acquiring a fibroblast-like cell shape, increased cell volume and cell protrusions [12,23,41]. Next, we investigated how OSL influenced the cell morphology of already diseased OA chondrocytes using box plots, which not only allows comparing treatment groups but also allows viewing the dispersion and spread of data on a large number of cells (Figure 3A–G). OSL dose-dependently and significantly decreased the area compared to control-treated cells by 3% and 11%, respectively (Figure 3A). Similarly, OSL dose-dependently decreased the cell’s major axis (cell length) by 3% and 13% (Figure 3B). Treatment with the lowest OSL concentration also significantly decreased the cell’s minor axis (cell width) by 2% vs. control-treated cells (Figure 3C). In line with this data, OSL dose-dependently decreased the aspect ratio (ratio of major to minor axis) by 3% and 13%, respectively (Figure 3E). Together, this demonstrates that OSL treatment caused the cells to become less elongated. This was compatible with OSL effects on circularity and roundness, which showed that OSL significantly and dose-dependently increased the cell’s circularity by 8% and 20% (Figure 3D) and roundness by 3% and 15% (Figure 3F) vs. control-treated cells. Similarly, OSL significantly and dose-dependently increased the solidity by 1% and 2% vs. control (Figure 3G), indicating few cell protrusions.

In summary, OSL treatment led to (i) dose-dependent effects on cell morphology and (ii) ameliorated the diseased shape of OA chondrocytes and reverted the cells to a healthy cell shape.

### 3.3. Positive and Negative Correlations between Measured Features under Treatment

**Figure 4:**
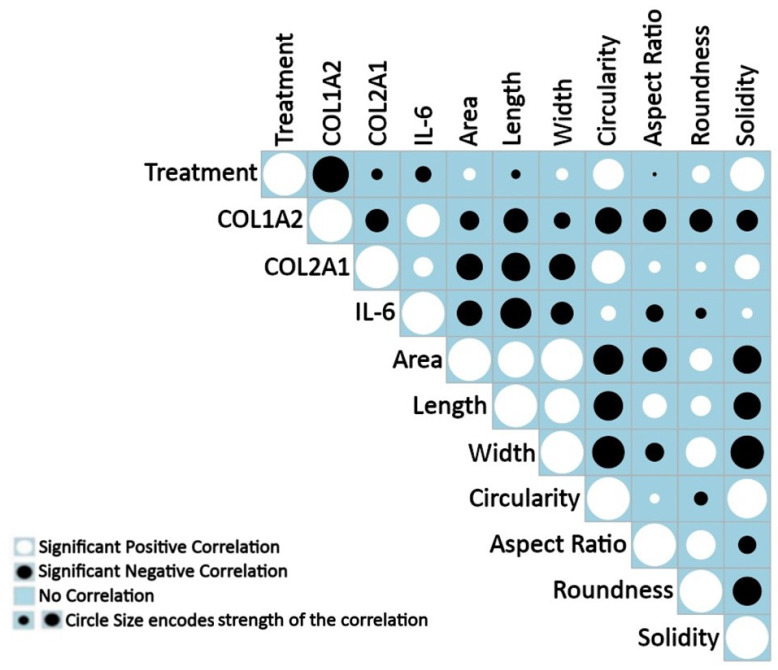
Correlograms depicting correlations in diseased cells under all conditions. Significant (*p* < 0.05) positive (white circles) or negative (black circles) correlations between features and the strength of the correlation is indicated by the size of the circle (larger circles indicate a stronger correlation having higher correlation coefficients). A blue empty box indicates a lack of correlation. Data is representative of the average gene expression and cell morphology values of 22,309 cells measured in the control, 20, and 80 µg/mL lignin-treated groups, respectively, for each of the cell morphology descriptors of n = 4 individual experiments.

As the next step, correlation analysis was performed to understand how lignin induced changes in human diseased OA morphology and phenotype and how these features related to one another under treatment (Figure 4). Importantly, all features significantly correlated suggesting a strong relationship between cell morphology and phenotype with lignin treatment. As expected, correlations among gene expression markers showed that COL1A2 expression negatively correlated with COL2A1 and positively with IL-6 expression. These findings agree with the known phenotype of OA chondrocytes (1). There was a strong (indicated by the correlation coefficient and the larger circle size) negative correlation between lignin treatment and the expression of COL1A2 in line with Figure 2 showing that lignin can significantly decrease its expression. There was also a negative correlation between lignin treatment and the expression of IL-6, supporting the idea that lignin can reduce IL-6. Surprisingly, there was a small negative correlation between lignin treatment and the expression of COL2A1. This suggested that some donors differed in their response to lignin as explained in more detail in the next section.

There was a strong significant positive correlation between lignin treatment and circularity, roundness and solidity, indicated by the correlation coefficient and the circle size in Figure 4, and a significant negative correlation with cell length showing that, similar to Figure 2, lignin treatment induced more circular, rounded and less fibroblastic-like cells and thereby induced a healthier cell morphology.

Importantly, the expression of the unhealthy COL1A2 marker significantly and strongly negatively correlated with all of the cell shape descriptors. In most cases, IL-6 followed a similar trend. The expression of COL2A1 significantly and strongly negatively correlated with area, length and width and positively with circularity and solidity. Collectively, this shows co-occurring morphological and phenotypical changes indicative of lignin-mediated modification of cell morphology and gene expression towards a less dedifferentiated and, hence, healthier morphology and phenotype.

### 3.4. Lignin-Mediated Modulation of the Gene Expression and Cell Morphology at the Sample Level

**Figure 5:**
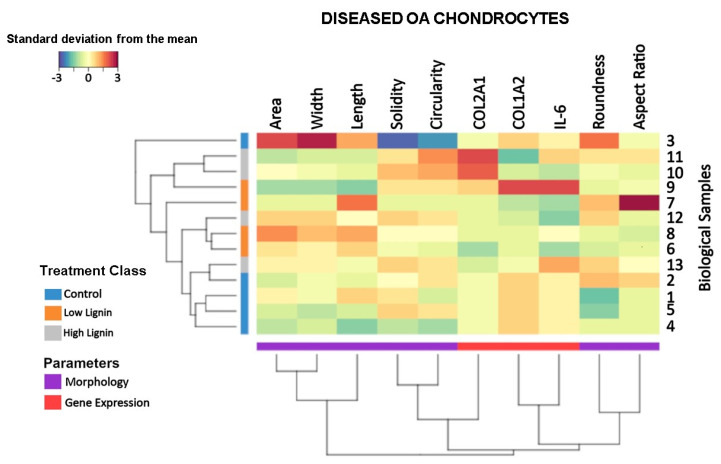
CIM plot demonstrating the effects of lignin on diseased OA chondrocytes at the sample level. The gene expression data from Figure 2 and the cell morphology data from Figure 3 was scaled and centered, allowing comparisons at the sample level using a clustered image map (CIM). The dendrograms cluster the biological samples based on parameter similarities. The scale on the upper left side of the figure describes the standard deviation below (blue) or above (red) the overall mean across all samples with the intensity representing increases and decreases of the measured feature from the overall mean. The samples were coded as follows: 0 (control-treated cells), 1 (low lignin = 20 µg/mL lignin-treated cells), and 2 (high lignin = 80 µg/mL lignin-treated cells).

As a final step, we used CIM, which allows multivariate data comparisons at the sample level (Figure 5). Hierarchical clustering was used because it allows viewing multivariate data over a variety of scales by creating a cluster tree or dendrogram with the height of the branch points indicating how similar or different the relationship between the entities is from one another: the greater the height, the greater the difference. This enabled us to explore relationships between linkages of clusters. As shown on the left side, with the exception of one of the controls (sample 3), the control samples clustered together. Treatment with low (20 µg/mL) lignin and high (80 µg/mL) lignin samples clustered into two and three groups, respectively, discussed in more detail below. As shown in the lower part of Figure 5, cell roundness and its inverse shape descriptor aspect ratio clustered, which we previously identified as key shape descriptors of fully diseased human OA chondrocytes and as early de-differentiating chondrocytes, induced by IL-1β [23]. COL1A2 and IL-6 gene expression also clustered demonstrating a relationship between these unhealthy fibrotic- and inflammatory-inducing markers. Whereas COL2A1 expression clustered with cell circularity and solidity, COL1A2 and IL-6 gene expression clustered with roundness and aspect ratio. This further demonstrates a close relationship between gene expression and cell morphology.

Using the CIM (the scale is shown in the upper left corner) allowed us to see how individual donors responded to lignin on the cell shape and gene expression level. Except sample 3, the control samples clustered together and generally showed the same trend under control treatment. As mentioned above lignin-treated samples clustered into several groups showing that, in some cases, individual samples differed in response to lignin. Importantly, with the exception of sample 9, all lignin-treated cells responded by decreasing COL1A2, the most important unhealthy marker of diseased OA chondrocytes and an important fibrotic-inducing gene. COL2A1, one of the most important healthy markers of chondrocytes and cartilage ECM, increased in two of four samples (samples 10 and 11) treated with the higher dose (80 µg/mL) of lignin. Lignin decreased IL-6 in four of eight samples (samples 6 and 8 treated with 20 µg/mL lignin and samples 10 and 12 treated with 80 µg/mL lignin). Overall, heat maps in samples 6, 8, 10 and 12 were relatively similar showing that regardless of dose, lignin modified the gene expression towards being healthier and less inflammatory while simultaneously regulating the cell shape into a healthier cell shape, which was more apparent using the higher dose of lignin. This demonstrates that low or high doses of lignin decreased COL1A2 expression in all donors but in half of the donors, low or high doses of lignin increased COL2A1 and decreased IL-6, which corresponds with a healthier cell shape.

## 4. Discussion

Despite the fact that lignin is the second most abundant global biopolymer on Earth [1], our understanding of how lignin modulates the cell morphology and function of diseased cells remains fragmentary. This is the first study to show that lignin modulated the cell morphology of human cells and concurrently decreased a major marker of disease, COL1A2. This pioneering work shows the therapeutic potential of lignin-mediated modulation of cell morphology and phenotype of diseased cells towards a healthier phenotype. Thus, lignin treatment led to (i) dose-dependent effects on cell morphology and (ii) ameliorated the diseased shape of OA chondrocytes by reverting the cells to a healthier cell shape, which correlated with positive changes in disease-, ECM- and inflammatory-regulating gene expression. This shows that lignin can change degenerative and inflamed diseased cells towards a healthier cell state.

While it is not known how lignin directly modulates the cell morphology of diseased human cells, we can extract from lignin’s role in nature and postulate how lignin could be capable of cell phenotype modulation. Lignin affects plant development by strengthening a plant’s robustness via adding a significant reinforcement to cell walls [6,7] as it does to tissue engineering cell scaffolds as we [16] and others [42,43,44,45] have shown. The mechanical stability that lignin provides to plants has recently been shown to correlate with lignin content and the cell morphology of plant cells [46]. We previously showed that other types of biomaterials, as well as their nanoscale surface stiffness and surface topography lead to significant changes in cell morphology and, importantly, large phenotypic effects [32,33,34,35]. The present study extends these findings and shows, for the first time, that lignin is a biomaterial that can modulate the cell morphology and phenotype of human cells. We previously showed that the low MW fraction of OSL used in this study had more numerous aliphatic hydroxyl functionalities, while having condensed phenolic structures and a less branched conformation as well as an increased hydrogen bonding capacity. This could hypothetically promote intermolecular interactions with the cells themselves or even the pericellular matrix of e.g., chondrocytes and potentially other cell types [16] and thereby modify the phenotype and function of cells towards a healthier phenotype as these characteristics would be expected to increase the availability of lignin’s functional sites and provide reinforcement. By reinforcing the cartilage pericellular matrix, OSL could also potentially enhance the mechanical properties of the artificial regenerative cartilage environment. This is important because the mechanical properties of cartilage play a critical role in its function and are often compromised in degenerative joint diseases. We have shown how the stiffness of the cartilage tissue degenerates in the progression of OA [47] and that tissue and biomaterial stiffness are key regulators of chondrocyte phenotype [48]. OSL could help to stabilize an artificial cartilage environment and help stabilize a chondrogenic phenotype.

Importantly, lignin significantly modified multi-factorial aspects of cell morphology including the area (decreased), length (decreased), width (decreased), roundness (increased), circularity (increased), solidity (increased), and the number of cytoplasmic processes (decreased). Thus, lignin induced a less fibroblastic cell shape, causing the cells to become more circular and less elongated. We previously showed that IL-1β caused early diseased chondrocytes to become morphologically and phenotypically more de-differentiated [23]. In the present study, we showed that lignin produced the opposite effect, causing diseased chondrocytes to become morphologically and phenotypically less de-differentiated. Therefore, lignin induced a less fibroblastic cell morphology and, therefore, a healthier chondrocyte shape, which correlated with a shift in phenotype from an unhealthy to a healthier cell state. Hence, lignin transformed the diseased cell morphology and shifted the diseased phenotype by significantly downregulating the expression of the unfavorable COL1A2, which leads to fibrotic ECM (fibrosis) and, in cartilage, a biomechanically instable weak ECM and joint instability [22]. Besides cartilage, the effect of lignin on the downregulation of COL1A2 may also be important in the regulation of other fibrotic diseases where the expression of type I collagen is increased such as in pulmonary, liver, and bone marrow fibrosis and scleroderma [49] or in tumor invasion and progression [50,51,52]. Moreover, in half of the donors, in addition to lignin-induced changes in cell morphology and inhibition of COL1A2 expression, treatment with the higher dose of lignin significantly upregulated the expression of COL2A1, which promotes a biomechanically-stable ECM and tissue homeostasis in cartilage [21]. It simultaneously downregulated the expression of IL-6, a cytokine deeply involved in uncontrolled inflammation in chronic inflammatory and autoimmune diseases, cancer, and the cytokine storm induced by viral diseases such as coronavirus disease 2019 (COVID-19) [20,53,54]. Together, this suggests novel therapeutic use of lignin to convert the cell morphology and phenotype of human diseased cells into healthy cells, which could benefit tissue engineering strategies but also control fibrosis and inflammation.

Lignin could be used alone (e.g., pharmaceutically) as a therapeutic modulator of diseased cells to therapeutically target cell function by altering diseased and inflamed cells towards a healthy status by modification of cell morphology and function. Lignin could also be used in a cell scaffold. We previously showed that the incorporation of organosolv lignin used in the present study significantly and dose-dependently increased scaffold stiffness and viscosity as well as chondrocyte cell attachment. Hansen solubility physiochemical parameters also showed high compatibility and interactive forces between lignin and agarose, demonstrating biocompatibility in a tissue engineering scaffold [16]. Moreover, the lignin and concentrations used in the present study were non-cytotoxic and biocompatible with other cell types including fibroblasts, MSCs, osteoblasts, and keratinocytes [16].

Therefore, incorporating lignin could improve tissue regeneration, e.g., in degenerative diseases related to cartilage such as cartilage defect repair, osteoarthritis, or intervertebral disc disease and, potentially, in other diseases and tissues as well via the effects shown in this study.

## 5. Conclusions

This is the first study to show that lignin can modulate many aspects of cell morphology and induce a healthier cell shape, which correlates with positive changes in ECM- and inflammatory-regulating genes and a concurrent decrease in a major marker of disease, COL1A2, in human cells. This study demonstrates that lignin can be exploited in an entirely new way, allowing the development of pharmaceutical and biomedical products that could give rise to versatile and innovative technologies by using lignin to therapeutically modulate cell morphology and phenotype of diseased cells towards a healthier cell state.

## Data Availability

The datasets used and/or analyzed during the current study are available from one of the corresponding authors upon reasonable request.
